# Modified multi-Rayleigh model-based statistical analysis of ultrasound envelope for quantification of liver steatosis and fibrosis

**DOI:** 10.1007/s10396-023-01354-3

**Published:** 2023-10-05

**Authors:** Yuki Ujihara, Kazuki Tamura, Shohei Mori, Dar-In Tai, Po-Hsiang Tsui, Shinnosuke Hirata, Kenji Yoshida, Hitoshi Maruyama, Tadashi Yamaguchi

**Affiliations:** 1 Graduate School of Science and Engineering, Chiba University, 1-33 Yayoicho, Inage, Chiba, 2638522 Japan; 2 Preeminent Medical Photonics Education and Research Center, Hamamatsu University School of Medicine, Hamamatsu, Shizuoka 4313192 Japan; 3 Graduate School of Engineering, Tohoku University, Sendai, Miyagi 9808579 Japan; 4 Department of Gastroenterology and Hepatology, Chang Gung Memorial Hospital at Linkou, Taoyuan, 33305 Taiwan; 5 Division of Pediatric Gastroenterology, Department of Pediatrics, Chang Gung Memorial Hospital at Linkou, Taoyuan, 33305 Taiwan; 6 Department of Medical Imaging and Radiological Sciences, College of Medicine, Chang Gung University, Taoyuan, 33305 Taiwan; 7 Center for Frontier Medical Engineering, Chiba University, 1-33 Yayoicho, Inage, Chiba, 2638522 Japan; 8 Department of Gastroenterology, Juntendo University, Bunkyo, Tokyo, 1138421 Japan

**Keywords:** Quantitative ultrasound, Amplitude envelope statistics, Multi-Rayleigh model, Chronic hepatitis, Fatty liver

## Abstract

**Purpose:**

Quantitative diagnosis of the degree of fibrosis progression is currently a focus of attention for fatty liver in nonalcoholic steatohepatitis (NASH). However, previous studies have focused on either lipid droplets or fibrotic tissue, and few have reported the evaluation of both in patients whose livers contain adipose and fibrous features. Our aim was to evaluate fibrosis tissue and lipid droplets in the liver.

**Methods:**

We used an analytical method combining the multi-Rayleigh (MRA) model and a healthy liver structure filter (HLSF) as a technique for statistical analysis of the amplitude envelope to estimate fat and fibrotic volumes in clinical datasets with different degrees of fat and fibrosis progression.

**Results:**

Fat mass was estimated based on the non-MRA fraction corresponding to the signal characteristics of aggregated lipid droplets. Non-MRA fraction has a positive correlation with fat mass and is effective for detecting moderate and severe fatty livers. Progression of fibrosis was estimated using MRA parameters in combination with the HLSF. The proposed method was used to extract non-healthy areas with characteristics of fibrotic tissue. Fibrosis in early fatty liver suggested the possibility of evaluation. On the other hand, fat was identified as a factor that reduced the accuracy of estimating fibrosis progression in moderate and severe fatty livers.

**Conclusion:**

The proposed method was used to simultaneously evaluate fat mass and fibrosis progression in early fatty liver, suggesting the possibility of quantitative evaluation for discriminating between lipid droplets and fibrous tissue in the early fatty liver.

## Introduction

Ultrasonography is used to observe and diagnose biological tissues such as those of the skin [1], liver [2], heart [3], and vascular system [4]. In addition to diagnostic methods based on ultrasound images, quantitative ultrasound (QUS) has long been proposed for the evaluation of the physical characteristics of biological tissue, based on echo signals [5]. QUS has been used to obtain amplitude envelope statistical properties [6–8], attenuation coefficients [9, 10], and backscatter coefficients [11, 12] related to the physical properties of biological tissue. Many researchers have reported the application of QUS to the abdominal region, particularly for investigation of the liver.

In recent years, the diagnosis of fatty liver, which is a chronic accumulation of lipid droplets in the liver, has been the focus of much attention in liver diseases. Magnetic resonance imaging proton density fat fraction (MRI-PDFF) is becoming the gold standard in fatty liver diagnosis [13]. In ultrasonography, a method to evaluate tissue-specific attenuation coefficients has been implemented in clinical settings [14, 15], and a positive correlation with fat mass evaluation has been obtained using MRI-PDFF [16]. In terms of amplitude envelope statistics, evaluation of fatty liver using the Nakagami distribution has been investigated in numerous studies [17]. However, the Nakagami model is not fitted well in the case of early fatty liver. For this reason, Tamura et al. proposed the double-Nakagami (DN) model, which comprises two components of the Nakagami model that have different parameters [18]. The healthy liver structure filter (HLSF) method was proposed to evaluate fat mass using the DN model parameters and performed well in clinical studies [19, 20].

In assessing hepatitis and cirrhosis, the degree of progression of liver fibrosis was evaluated using the multi-Rayleigh (MRA) model [21–23] as a method for assessing the amplitude envelope statistics of the echo signals from multiple scatterers. The parameters of the MRA model were calculated from moments that characterize the amplitude envelope probability distribution of the echo signal. Fibrotic liver has an inhomogeneous structure containing highly echoic fibrotic tissue and nodules of hypoechoic tissue. In the case of advanced fibrosis, it is known that the first-order and third-order moments are low and high, respectively, and change with the degree of fibrosis progression [21]. Shear wave elastography (SWE), which is used to assess stiffness based on the velocity of shear waves propagating through the tissue [24, 25], has also been incorporated into clinical instruments [26].

In the above examples, progression of either steatosis or fibrosis is the target. However, as the number of patients with nonalcoholic steatohepatitis (NASH) increases, the degree of liver fibrosis in fatty liver has become an essential factor in determining patient prognosis [27]. When the above evaluation methods based on SWE and attenuation coefficient are applied to the NASH liver, it is difficult to discriminate between the two tissue types because the acoustic properties of both fibrous tissue and lipid droplets are obtained [28]. In addition, diagnosing cases of early fibrosis of fatty liver is difficult and requires further validation [29, 30].

In this study, we performed a quantitative evaluation of the structure of NASH liver, which has both fibrotic and fatty characteristics. The HLSF method and MRA model were used independently to evaluate the characteristics of fibrosis and fat. There are several methods of envelope statistical analysis including HLSF, which has been proposed for fatty liver structures with the DN model. Fundamental to HLSF is two-dimensional QUS parameter evaluation using QUS parameters acquired with an echo dataset of healthy livers. In the present study, we adapted HLSF to MRA model parameters to evaluate the progression of fibrosis. The envelope amplitude distribution of aggregated lipid droplets is presumed to deviate significantly from the Rayleigh distribution. In this study, the criterion for tissue discrimination was whether or not the lipid droplets were judged as non-Rayleigh components. We applied these signal processing techniques to clinical data of livers having different degrees of fibrosis and lipid progression. There are two novel aspects of the present paper. First, it applies HLSF to MRA model parameters. Second, it attempts to quantify fibrosis and steatosis simultaneously by applying the MRA component count and HLSF methods to the same clinical data.

## Materials and methods

### Clinical echo dataset

The echo dataset used was of 204 patients with chronic hepatitis B or C. All patients had been diagnosed with progression of fibrosis and steatosis based on liver biopsy or partial hepatectomy. Table 1 shows the number of patients each with fibrosis and steatosis (fibrosis and steatosis groups). Fibrosis progression was evaluated using METAVIR score and classified into five levels of fibrosis: F0 (no fibrosis), F1 (portal fibrosis without septa), F2 (portal fibrosis with few septa), F3 (numerous septa without cirrhosis), and F4 (cirrhosis) [31]. Steatosis progression focused on the ratio of intracellular fatty deposition as a percentage and was classified into four grades of fatty liver: S0 < 5%, 5% ≦ S1 < :33%, 33% ≦ S2 < 66%, S3 ≧ 66%. The histological samples were examined at the Department of Pathology at Chang Gung Memorial Hospital, Linkou, Taiwan. All patients underwent abdominal ultrasound examination performed by a radiologist at Chang Gung Memorial Hospital. A clinical ultrasound scanner (Model 3000; Terason, Burlington, USA) equipped with a convex-array probe (Model 5C2A, Terason) with a center frequency of 3 MHz was used for the acquisition of radiofrequency (RF) data. The center frequency of the measurement system is lower than that used for abdominal diagnostics in recent years. The transducer has a pulse length of approximately 2.3 mm [32]. During data acquisition, the focal length and maximum depth were fixed at 40 mm and 80 mm, respectively. The sampling frequency of the measurement system was 30 MHz, and 128 scan lines were obtained in cross section.

**Table 1:** Classification of fibrosis and steatosis progression based on histopathological findings

	METAVIR score					Total
	F0	F1	F2	F3	F4	
Fatty liver grade						
S0	11	10	24	21	14	80
S1	2	18	14	23	13	70
S2	0	6	8	13	9	36
S3	3	6	1	3	5	18
Total	16	40	47	60	41	204

### Amplitude probability distribution model

The Rayleigh distribution is the probability density distribution of the amplitude envelope of echo signals from a homogeneous medium that has a dense distribution of scatterers [33]. The probability density function (PDF) of the Rayleigh distribution is given as

1 $$ p_{{{\text{RA}}}} \left( x \right) = \frac{2x}{{\sigma^{2} }}{\text{exp}}\left[ { - \frac{{x^{2} }}{{\sigma^{2} }} } \right], $$

where $x$ is the amplitude envelope of the echo signal and ${\sigma }^{2}$ is the echo signal energy. In the case of normal liver, the probability density distribution of the amplitude envelope of echo signals is the Rayleigh distribution because of the arrangement of luminal structures in a regular pattern as the main sources of scattering.

However, livers with advanced disease such as cirrhosis and NASH have mixed fibrous tissue and lipid droplets in addition to luminal structures as the scattering sources. Therefore, liver with advanced disease has a mixed tissue structure comprising multiple types of scattering sources with different intrinsic acoustic properties and sizes. Accordingly, the PDF of the amplitude envelope deviates from the Rayleigh distribution. As the acquired echo signal is expressed as the sum of signals from each scatterer, the amplitude envelope probability density deviates from the Rayleigh distribution. Therefore, an MRA model that can discriminate the signals from multiple scatterers was proposed, to mainly use to evaluate the degree of progression of liver fibrosis [21–23]. The MRA model with three components is given as

2 $$ p_{{{\text{MRA}}}} \left( x \right) = \alpha_{{\text{L}}} p_{{\text{L}}} \left( x \right) + \alpha_{{\text{M}}} p_{{\text{M}}} \left( x \right) + \alpha_{{\text{H}}} p_{{\text{H}}} \left( x \right), $$

where $p_{{\text{L}}}$, $p_{{\text{M}}} ,$ and $p_{{\text{H}}}$ are the Rayleigh distributions with different scale parameters $\sigma_{{\text{L}}}^{2}$, $\sigma_{{\text{M}}}^{2}$, and $\sigma_{{\text{H}}}^{2}$, respectively. In Eq. (2), the scale parameters are constrained to be $\sigma_{{\text{L}}}^{2} < \sigma_{{\text{M}}}^{2} < \sigma_{{\text{H}}}^{2} .$ $\alpha_{{\text{L}}}$, $\alpha_{{\text{M}}}$, and $\alpha_{{\text{H}}}$ are the mixture rate of each Rayleigh distribution, which can be expressed as the amount of corresponding tissue. Because $p_{{{\text{MRA}}}}$ is the probability density function of the MRA model, the relationship of the mixture rate is $\alpha_{{\text{L}}} + \alpha_{{\text{M}}} + \alpha_{{\text{H}}} = 1$. Therefore, $p_{{\text{M}}}$ represents the signal component from normal liver tissue, $p_{{\text{L}}}$ represents hypoechoic tissue or structures with a sparse scatterer distribution, and $p_{{\text{H}}}$ represents hypoechoic tissue or tissue with a dense scatterer distribution. In the case of fibrous liver, each parameter is assumed to correspond to nodules, normal liver tissue, and fibrotic tissue, respectively. Focusing on fibrotic tissue, the parameter $\alpha_{{\text{H}}}$ corresponds to the fibrotic tissue mixing ratio and $\sigma_{{\text{H}}}^{2} /\sigma_{{\text{M}}}^{2}$ is the echo intensity ratio of fibrotic tissue to normal liver tissue. To evaluate fibrosis progression in the liver, $\alpha_{{\text{H}}}$ and $\sigma_{{\text{H}}}^{2} /\sigma_{{\text{M}}}^{2}$ were used in combination as fibrosis parameters.

Note that during the analysis, the amplitude envelope is normalized so that $E[x^{2} ] = 1$. Since the analysis is normalized to the second moment of the amplitude envelope, the parameters of the MRA model satisfy Eq. (3):

3 $$ \alpha_{{\text{L}}} \sigma_{{\text{L}}}^{2} + \alpha_{{\text{M}}} \sigma_{{\text{M}}}^{2} + \alpha_{{\text{H}}} \sigma_{{\text{H}}}^{2} = 1. $$

The parameters of each component of the MRA model are estimated from the above condition.

### Estimation of MRA parameters by estimation of number of Rayleigh components

In the amplitude envelope statistics, the ROI was 5.7 × 7.2 mm (depth × lateral direction) in the scan-converted B-mode image, which is 3 × 3 times the point spread function (PSF) of the clinical scanner. We selected the area for analysis within the liver parenchyma manually, excluding apparent blood vessels. The ROI for analysis was set so that the actual physical size was constant at any location within the analysis area.

**Fig. 1:**
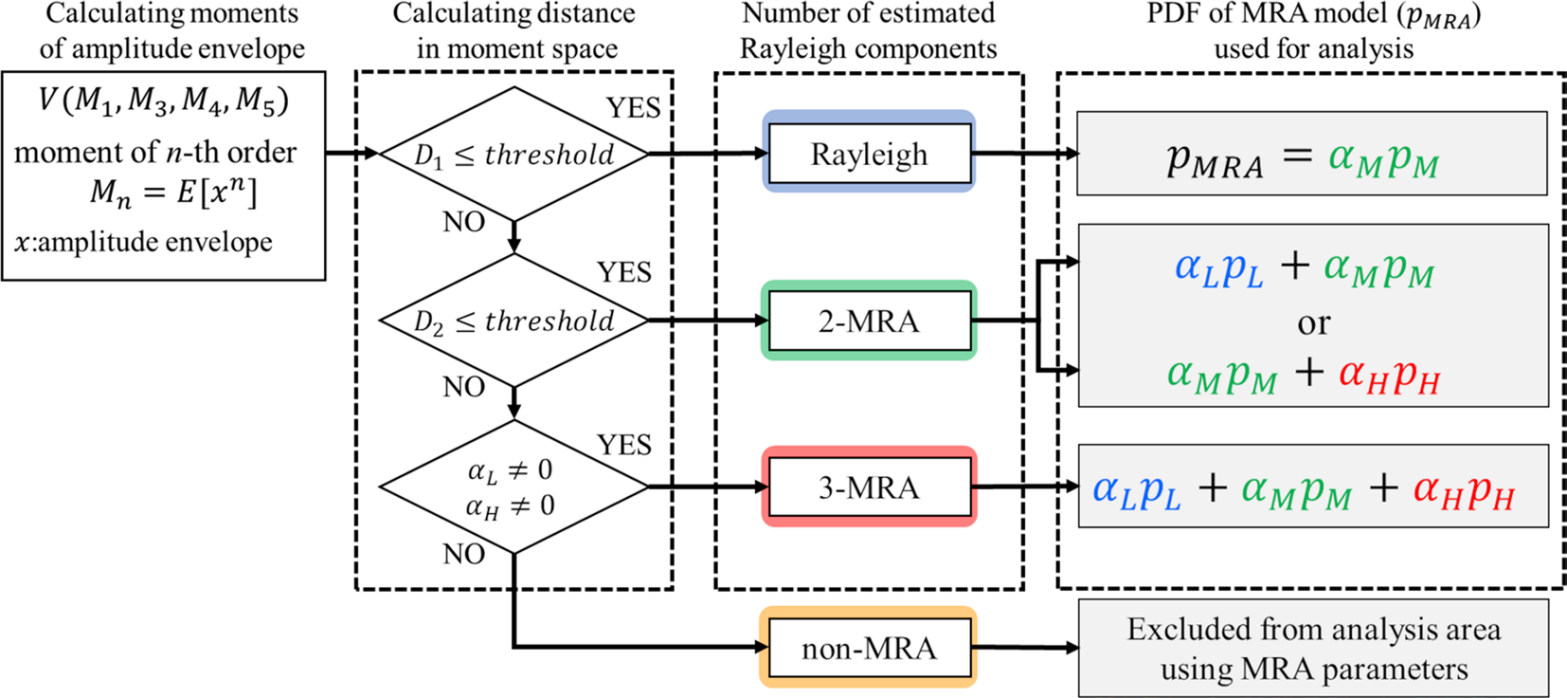
Conceptual diagram of flow for determining the number of components in the MRA model

The parameters of the MRA model were estimated for each scanned ROI. First, the MRA model estimated the number of Rayleigh components used for evaluation. The number of components of the Rayleigh distribution was determined from the statistical properties of the evaluation signal. In a previous study, Mori et al. proposed a method for estimating the number of tissues using moments of the amplitude envelopes [23]. The present study uses the first-, third-, fourth-, and fifth-order moments to estimate the number of Rayleigh components based on the squared Mahalanobis distance in moment space from the theoretical moments of Rayleigh distribution and the MRA model with two components. Figure 1 shows a conceptual flow diagram for estimating the number of components in the MRA model. In Fig. 1, ${D}_{1}$ is the distance from the theoretical moments of Rayleigh distribution. Since the squared Mahalanobis distance follows a Chi-square distribution, the threshold for the one-component determination was set at 9.49 based on the cumulative probability density distribution of the Chi-square distribution. If the value of ${D}_{1}$ is higher than 9.49, the threshold of Rayleigh distribution, then ${D}_{2}$ is calculated as the distance from the theoretical moments of the MRA model with two components (2-MRA). The threshold value for the two-component multi-Rayleigh model was set at 4.53 based on the mean value of the cumulative probability density distribution of the squared Mahalanobis distance for each parameter combination. If ${D}_{2}$ is higher than 4.53, the threshold of the 2-MRA model, an MRA model with three components (3-MRA) is used for evaluation. In the basic study using a tissue-mimicking phantom, it was found that the estimation of the number of Rayleigh components worked as well as the probability in the theoretical method [34]. The method of estimating the number of Rayleigh components based on distance in moment space does not depend on differences in measurement systems, and it is possible to determine a threshold such that the statistical probability of correctly estimating the number of tissue components is constant. In ROIs determined to be 2-MRA, the 2-MRA combination is determined based on the relationship between the scale parameter (${\sigma }_{\mathrm{M}}^{2}$) of the one- and three-component estimated analysis points in the ROI and the parameters. For explanation, the scale parameters of the 2-MRA model before determining the two-component combination are defined to be $\hat{\sigma }_{{\text{L}}}^{2}$ and $\hat{\sigma }_{{\text{H}}}^{2}$ ($\hat{\sigma }_{{\text{L}}}^{2} < \hat{\sigma }_{{\text{H}}}^{2}$). When the tissue is composed of two components, there are two cases of tissue composition, (hypoechoic tissue + normal tissue) and (normal tissue + fibrotic tissue). Since it can be assumed that the power of the echo signal from normal liver tissue does not change in the nearby region, the tissue composition of the 2-MRA model can be obtained by comparing it with the echo signal power ($\sigma_{{\text{M}}}^{2}$) from normal liver tissue in the near field of the ROI. In the case of $\left| {\hat{\sigma }_{{\text{L}}}^{2} - \sigma_{{\text{M}}}^{2} } \right| > \left| {\hat{\sigma }_{{\text{H}}}^{2} - \sigma_{{\text{M}}}^{2} } \right|$, the combination of the 2-MRA model is ($\alpha_{{\text{L}}} p_{{\text{L}}} + \alpha_{{\text{M}}} p_{{\text{M}}}$), because $\hat{\sigma }_{{\text{H}}}^{2}$ corresponds to the power of normal liver tissue. On the other hand, if $\left| {\hat{\sigma }_{{\text{L}}}^{2} - \sigma_{{\text{M}}}^{2} } \right| < \left| {\hat{\sigma }_{{\text{H}}}^{2} - \sigma_{{\text{M}}}^{2} } \right|$, the combination of the 2-MRA model is ($\alpha_{{\text{M}}} p_{{\text{M}}} + \alpha_{{\text{H}}} p_{{\text{H}}}$). The tissue combination ($\alpha_{{\text{L}}} p_{{\text{L}}} + \alpha_{{\text{M}}} p_{{\text{M}}}$) or ($\alpha_{{\text{M}}} p_{{\text{M}}} + \alpha_{{\text{H}}} p_{{\text{H}}}$) of the 2-MRA model was determined by comparing the echo intensity of normal liver tissue at the analyzed points in the ROI [23].

In estimation of the number of Rayleigh components, we found a case in which the parameter estimation result showed one component (Rayleigh distribution) with ${\alpha }_{\mathrm{L}}=0$ and ${\alpha }_{\mathrm{H}}=0$, even if the estimation was determined to be 3-MRA. In this case, the moments of probability density of the amplitude envelope have a higher value of the first moment and a lower value of the third moment compared to the theoretical value of the Rayleigh distribution, which is significantly different from the characteristics of fibrotic tissue moments. We termed this characteristic non-MRA and excluded it from the evaluation area using the MRA model parameters. The characteristics of this moment are similar to those of aggregated lipid droplets, and it is considered to be a state that includes a coherent signal component that is not assumed in the multi-Rayleigh model. Therefore, we defined the non-MRA fraction as the percentage of the analysis area that was determined to be non-MRA and used this parameter as an indicator of the characteristics of lipid droplets.

### MRA model parameter evaluation with HLSF

**Fig. 2:**
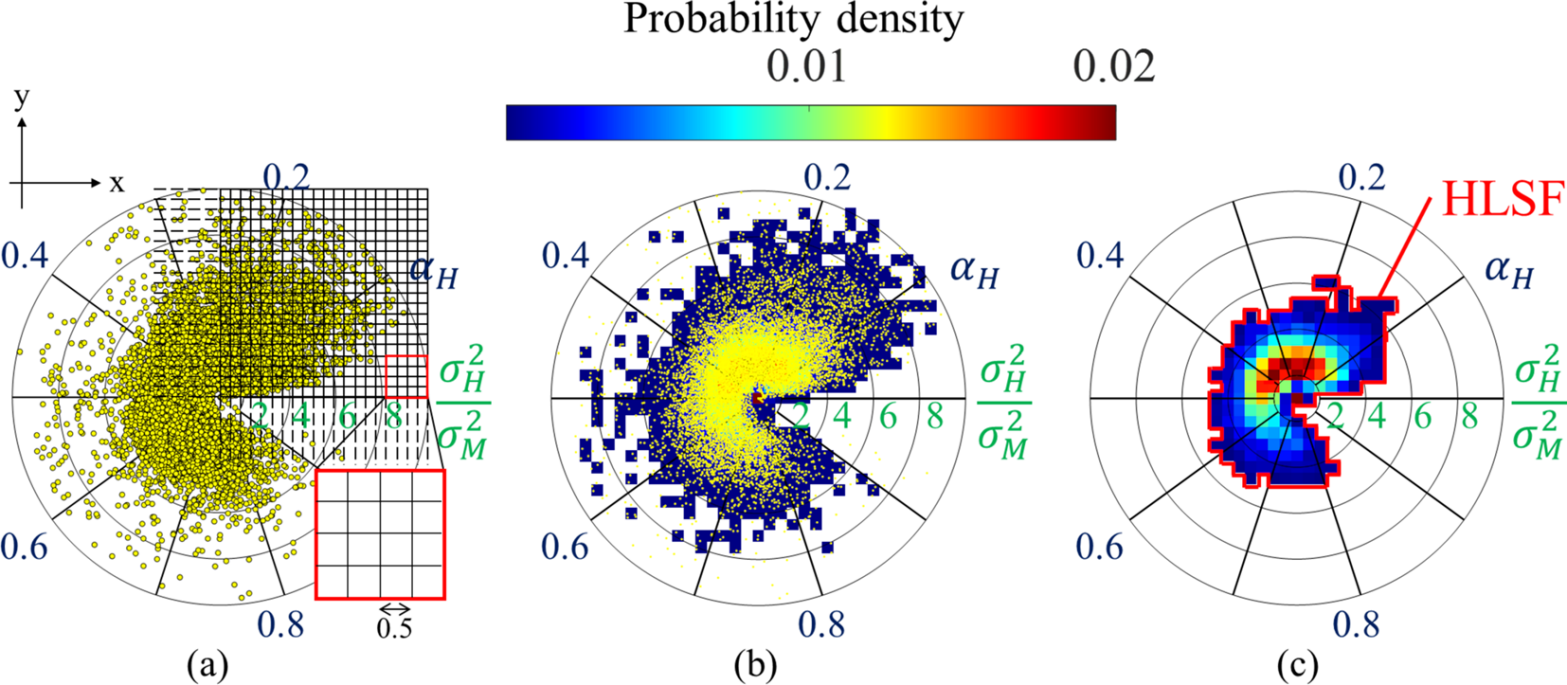
Conceptual diagram showing generation of the HLSF. **a** Scatter plot of all ROIs of livers classified as both S0 and F0 (*n* = 11). **b** Two-dimensional probability density distribution of the fibrosis parameters. **c** HLSF is determined based on the average distribution in healthy livers. Red color indicates the HLSF

To extract the region with non-healthy tissue features, a new analysis method was proposed based on the HLSF method combined with the MRA model parameters. The HLSF method classifies whether the ROI contains healthy liver structures [19]. In the MRA model, the fibrosis parameters $\alpha_{{\text{H}}}$ and $\sigma_{{\text{H}}}^{2} / \sigma_{{\text{M}}}^{2}$ are represented using a polar coordinate system, and a method has been proposed to evaluate both parameters simultaneously. Previous studies have shown that when the MRA model functions ideally, $\alpha_{{\text{H}}}$ corresponds to the amount of fibrous tissue in the ROI and $\sigma_{{\text{H}}}^{2} / \sigma_{{\text{M}}}^{2}$ to the dispersion ratio of fibrous tissue [23]. In the present study, the HLSF was defined from the distribution of the fibrosis parameters based on the MRA model. A diagram of the HLSF method is shown in Fig. 2. First, the MRA model parameters $\alpha_{{\text{H}}}$ and $\sigma_{{\text{H}}}^{2} / \sigma_{{\text{M}}}^{2}$ of all livers of S0 and F0 (of 11 patients) were estimated and plotted with polar coordinates using Eqs. (3) and (4) (Fig. 2a). The angle $\theta$ varied from 0 to $2\pi$ rad when $\alpha_{{\text{H}}}$ ranged from 0 to 1 in Eq. (3). The radial distance $r$ was defined as the echo signal power ratio of fibrotic tissue $\sigma_{{\text{H}}}^{2}$ to normal liver tissue $\sigma_{{\text{M}}}^{2}$ in Eq. (4). The MRA model parameters were plotted as yellow points in normal livers in Fig. 2a:

3 $$ \theta = 2\pi \alpha_{{\text{H}}} , $$

4 $$ r = \sigma_{{\text{H}}}^{2} /\sigma_{{\text{M}}}^{2} - 1. $$

A rectilinear grid with a grid spacing of 0.5 is shown in the *x*- and *y*-directions, and the number of ROIs within each square region calculated. Second, the number of ROIs in each square region was divided by the number of ROIs in normal livers and converted into a probability density. Figure 2b shows an example of normal liver in which the ROIs were converted into a two-dimensional probability density distribution. Finally, to acquire the distribution characteristics of the average fibrosis parameters in the normal livers, the region with the upper 90% probability of the two-dimensional probability density distribution was extracted and defined as the HLSF (Fig. 2c).

Sample MRA model parameters were plotted at the same polar coordinates. All MRA model parameters, the number of total plotted points (${N}_{\mathrm{total}}$), and the number of plotted points outside of the HLSF (${N}_{\mathrm{outside}}$) were then plotted. The ratio of ${N}_{\mathrm{outside}}$ to ${N}_{\mathrm{total}}$ was defined as the non-healthy area fraction (${\mathrm{Frac}}_{\mathrm{non}-\mathrm{healthy}}$). In calculating ${\mathrm{Frac}}_{\mathrm{non}-\mathrm{healthy}}={N}_{\mathrm{outside}}$/${N}_{\mathrm{total}}$, combinations of fibrosis parameters with extremely low probability densities (< 0.001) were excluded.

### Statistical analysis

Analysis of variance (ANOVA) was performed for fatty liver (four non-MRA grades) and fibrosis (five levels) to identify statistically significant differences in the acquired parameters between the grades. The evaluation indices were non-MRA fraction and non-healthy area fraction. If a significant difference was confirmed in this test, a two-group multiple comparison test was performed to examine the two groups with significant differences. Spearman correlations analysis was performed to evaluate the relationship between the evaluation indices (non-MRA fraction and non-healthy area fraction) and the fat mass of the patients. For all tests, a probability of less than 0.05 was considered statistically significant. All statistical analyses were performed in MATLAB (2021a, Math Works).

## Results

### Relationship between non-MRA and fat content

**Fig. 3:**
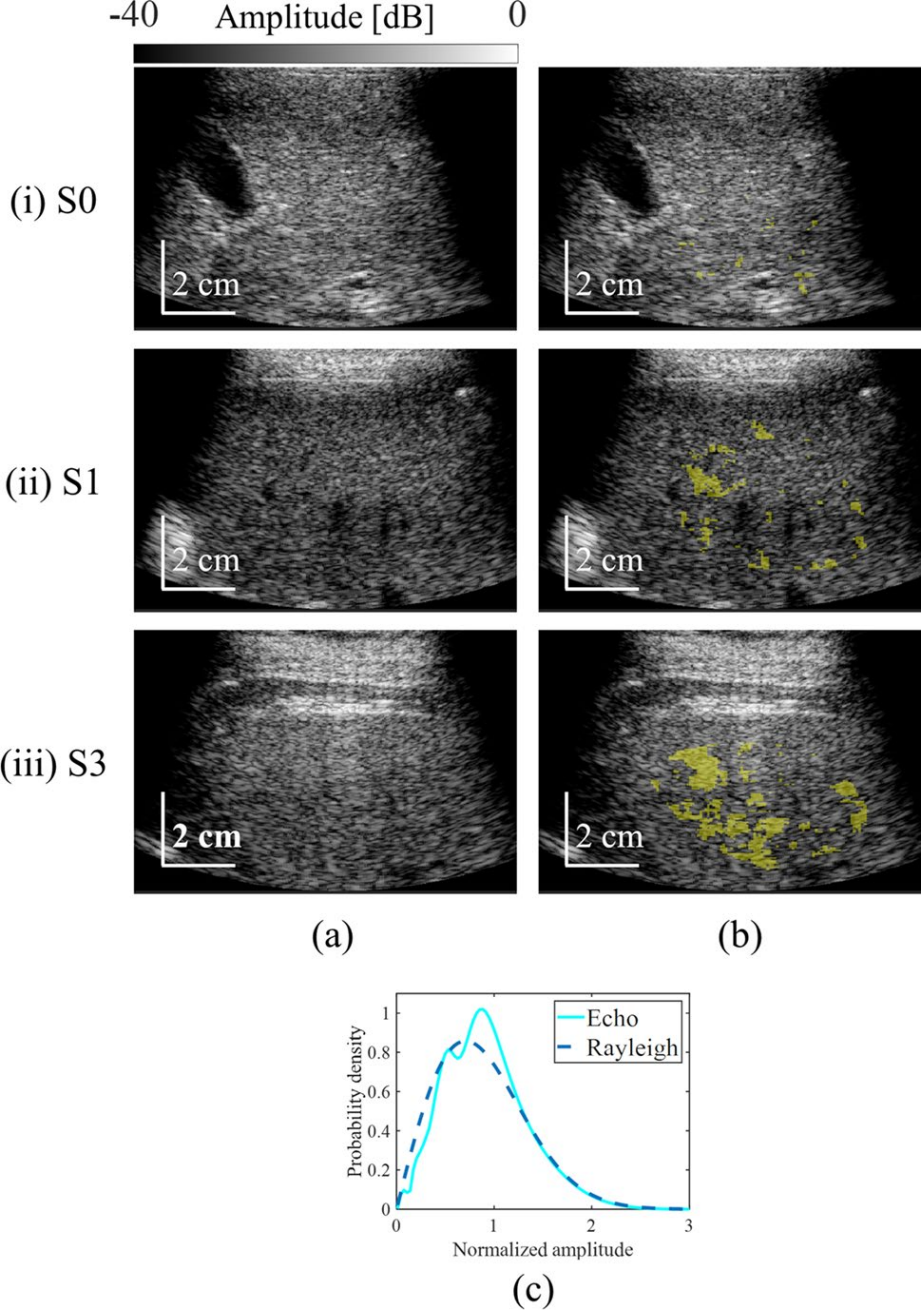
Non-MRA parameter mapping on B-mode images and probability density of echo amplitude in non-MRA. B-mode images (**a**) and parametric images (**b**) show the area judged as non-MRA. Images (i), (ii), and (iii) were obtained in patients graded as having F0 and S0 (0%), S1 (30%), and S3 (90%) fatty liver, respectively. Representative probability density of echo amplitude is shown for areas classified as non-MRA (**c**)

Figure 3 shows representative non-MRA parameter maps on B-mode images and the probability density of echo amplitude. Figure 3a–c show B-mode images, parametric images of non-MRA area, and the probability density of echo amplitude and Rayleigh distribution in the non-MRA area. In Fig. 3, (i), (ii), and (iii) are S0, S1, and S3, respectively, in F0 livers (a) and (b). The non-MRA fraction was 1.7% (S0), 11.0% (S1), and 26.0% (S3). Figure 3c, a representative PDF of the region determined to be non-MRA, appeared to be a composition of two Rayleigh distributions in shape. In actual evaluation results, however, it did not fit at all as a composite signal of two or three Rayleigh distributions, and one Rayleigh distribution came closest. Nevertheless, the large deviation from a single Rayleigh distribution was due to the inclusion of the aforementioned coherent component in the analysis domain.

The representative PDF of the region judged as non-MRA deviated from the Rayleigh distribution (Fig. 3c).

**Fig. 4:**
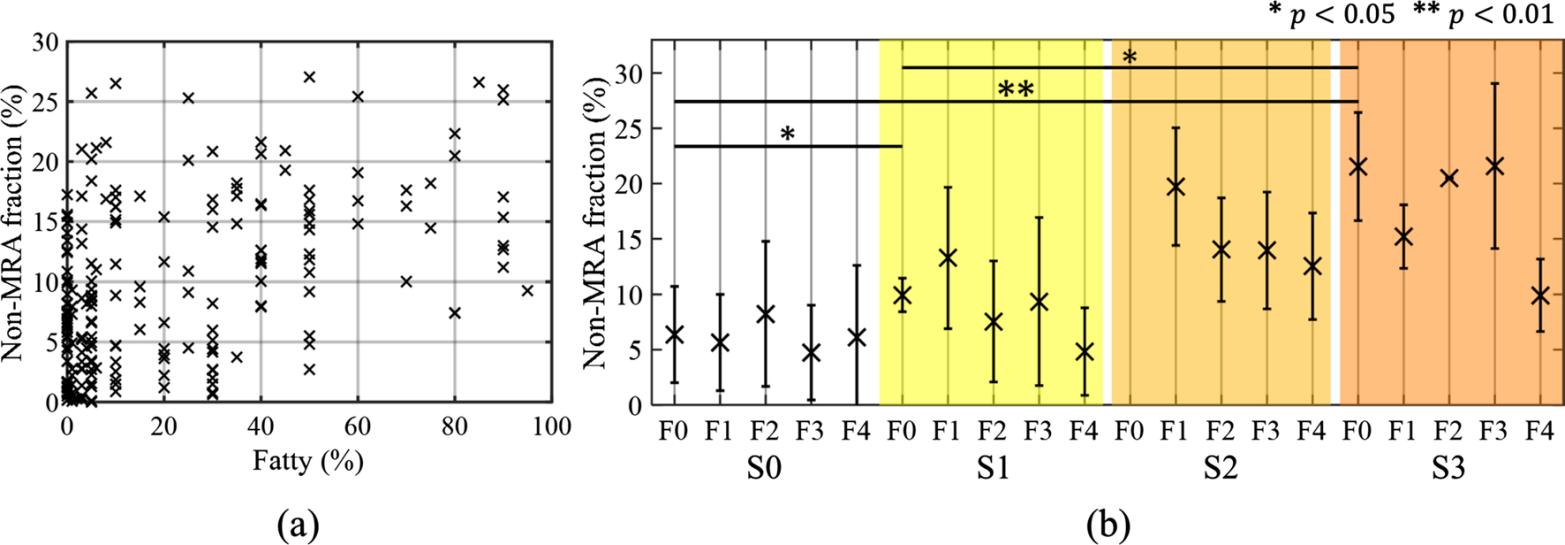
Relationship between non-MRA fraction and fat content in all livers (**a**) and mean values and standard deviation of non-MRA fraction for each fatty liver grade and level of fibrosis progression (**b**). In the comparison of non-MRA percentages for each fatty liver grade and fibrosis progression, there were statistically significant differences between S0 and S1 (*$p<0.05$) and S0 and S3 (**$p<0.01$), and between S1 and S3 (*$p<0.05$) for F0 liver in multiple comparison tests

The relationship between non-MRA fraction and steatosis progression was evaluated. Figure 4a shows the fraction of non-MRA in all livers. The non-MRA fraction had a positive correlation ($r=0.458, p<0.001$) with steatosis progression. In particular, livers with fat mass > 33% had higher values of non-MRA fraction compared with those with fat mass < 5%. However, in a few livers with fat mass < 5%, the non-MRA fraction exceeded 20%, which was an equivalent value to livers with fat mass > 33%. Figure 4b shows the mean value and standard deviation of the non-MRA fraction for each fatty liver grade (S0–S3) and fibrosis progression (F0–F4). In S0, there was no change in non-MRA fraction as fibrosis progressed. However, the non-MRA fraction decreased as fibrosis progressed in S1–S3. In addition, in S1–S3, the mean value of non-MRA fraction for each grade of fibrosis progression was 8.5% higher than F0 in S1, 11.6% lower than F0 in S3, and 7.2% lower than F1 in S2. The non-MRA fractions in S2 and S3 were approximately twice those of S0 at the same levels of fibrosis progression. Compared among F0 livers without fibrosis, the non-MRA fraction increased with steatosis progression: S0 (6.4%), S1 (10.0%), and S3 (21.5%). The non-MRA fraction in S0 livers was lower than those in S1 and S3. Analysis of variance (ANOVA) confirmed a significant difference ($p<0.001$); multiple comparison test of the two groups showed that the non-MRA fraction of the S0 and F0 liver was significantly different than that of S1 ($p=0.021$) and S3 ($p<0.001$) in F0 livers. No significant difference in non-MRA fraction with fatty liver progression was observed in patients with fibrosis progression F3 and F4.

### Evaluation of fibrosis progression in MRA parameters

**Fig. 5:**
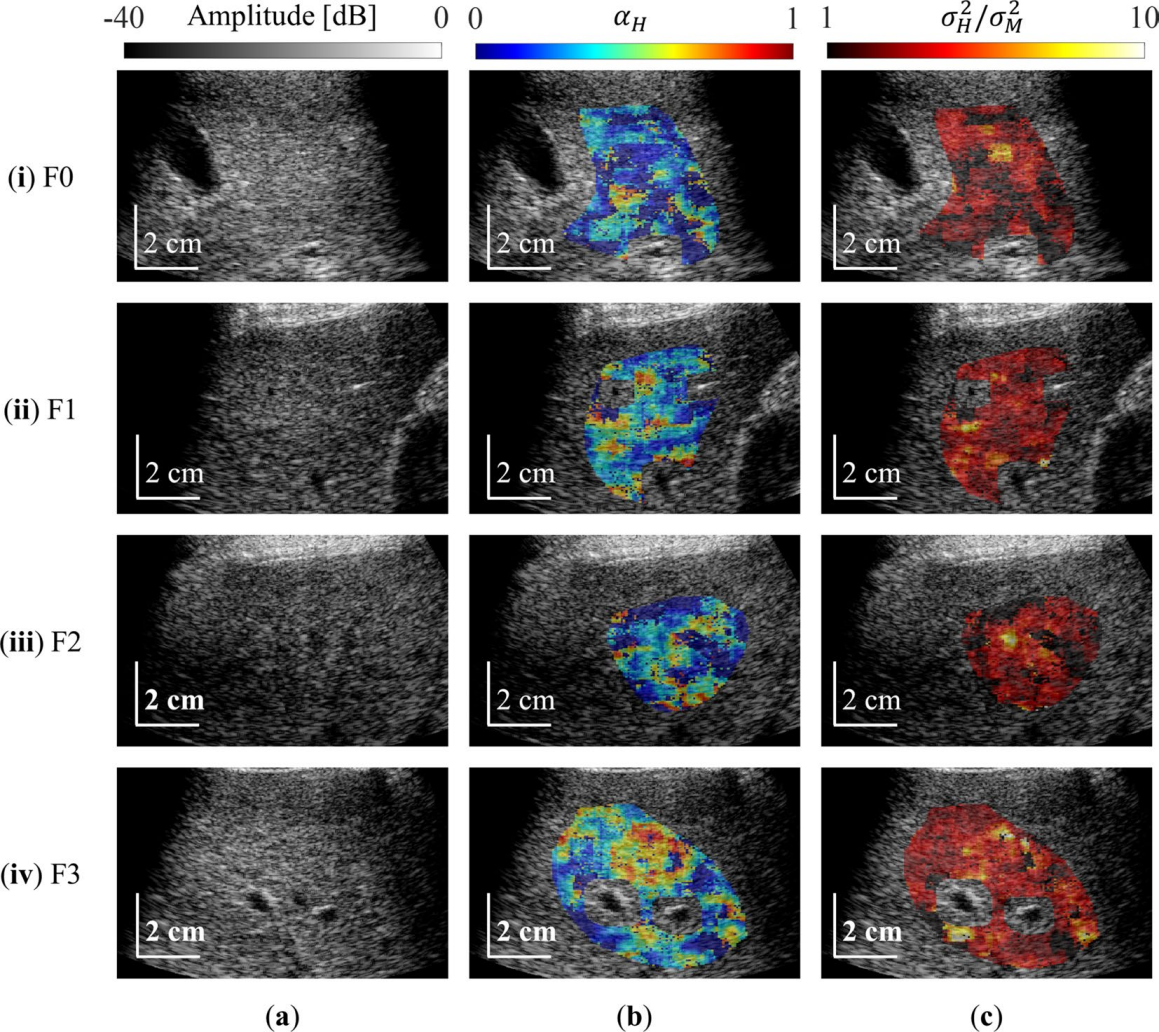
Representative results of MRA parameter mapping on B-mode images for grades F0–F4 in S0 livers. B-mode images (**a**), parametric images of ${\alpha }_{\mathrm{H}}$ (**b**), and parametric images of ${\sigma }_{\mathrm{H}}^{2}/{\sigma }_{\mathrm{M}}^{2}$ (**c**) are shown. The patients were classified as (i) F0, (ii) F1, (iii) F2, or (iv) F3 based on METAVIR score

Figure 5 shows representative MRA parameter maps on B-mode images for grades F0–F4 in S0 livers. There was no significant change in the appearance of B-mode images in terms of level of fibrosis progression (Fig. 5a). As shown in Fig. 5b, $\alpha_{{\text{H}}}$ varied from 0 to 1. In F3, there was a slightly greater area with high $\alpha_{{\text{H}}}$ values (i.e., level 1, shown in red) compared with that in F0. Figure 5c shows $\sigma_{{\text{H}}}^{2} /\sigma_{{\text{M}}}^{2}$. In F0, these values were mainly low (i.e., 1, in black), whereas the values were higher in F4 (i.e., 10, yellow region). Figure 5a, b shows that the area where the fibrosis parameter was estimated to be high increased with the progression of fibrosis.

**Fig. 6:**
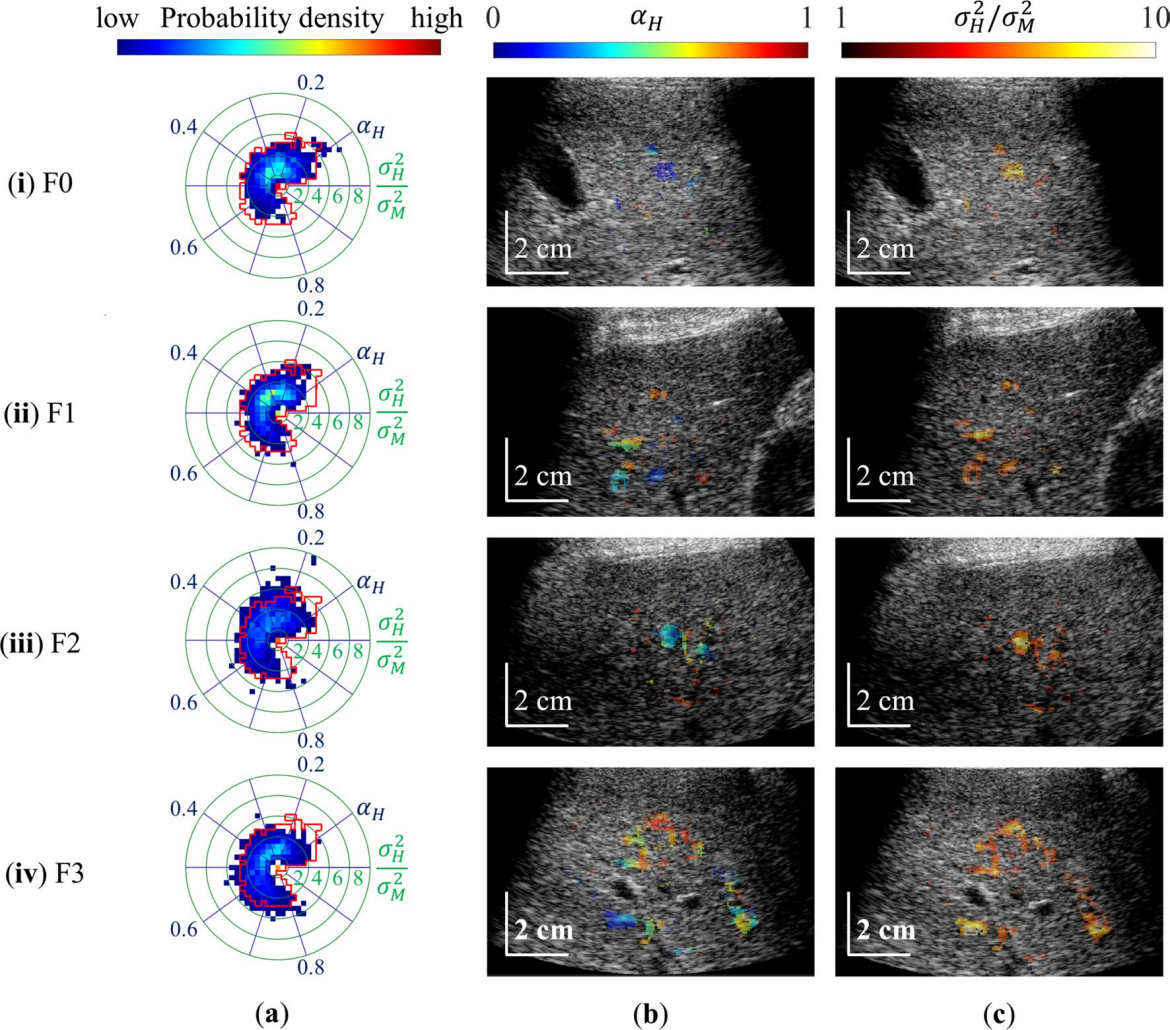
Non-healthy liver features extracted using HLSF. Representative B-mode images are shown for grades F0–F3 in S0 livers, in the same patients as in Fig. 4b. **a** Two-dimensional probability density distribution of the fibrosis parameters ${\alpha }_{\mathrm{H}}$ and ${\sigma }_{\mathrm{H}}^{2}/{\sigma }_{\mathrm{M}}^{2}$ with the polar coordinate system. Red lines show the edge of HLSF. **b**, **c** Parametric mapping of ${\alpha }_{\mathrm{H}}$ and ${\sigma }_{\mathrm{H}}^{2}/{\sigma }_{\mathrm{M}}^{2}$ extracted using the HLSF method

Figure 6 shows probability density distributions of the MRA parameters and displays non-healthy areas extracted using the HLSF for grades F0–F4 in S0 livers. Figure 6a shows probability distributions of fibrosis parameters using polar coordinates. In the polar coordinates of Fig. 6a, the angular direction corresponds to ${\alpha }_{\mathrm{H}}$ and the radial direction to ${\sigma }_{\mathrm{H}}^{2}/{\sigma }_{\mathrm{M}}^{2}$. The solid red line represents the HLSF, and the area outside the HLSF represents the non-healthy area. The distribution of fibrosis parameters showed a tendency to spread in the radial direction as fibrosis progressed. In the case of F3, high values were found for the echo intensity ratio of fibrotic tissue to normal liver tissue $\sigma_{{\text{H}}}^{2} /\sigma_{{\text{M}}}^{2}$, and the probability distribution of fibrosis parameters expanded beyond HLSF. The non-healthy area fractions were 5.0% (F0), 9.4% (F1), 11.0% (F2), and 16.7% (F3). Figure 6b shows parametric images of $\alpha_{{\text{H}}}$ extracted using the HLSF. The $\alpha_{{\text{H}}}$ values were not constant in the non-healthy area. However, local $\alpha_{{\text{H}}}$ values were higher in F3 compared with F0. Figure 6c shows parametric images of $\sigma_{{\text{H}}}^{2} /\sigma_{{\text{M}}}^{2}$ extracted using the HLSF. In contrast to $\alpha_{{\text{H}}}$, $\sigma_{{\text{H}}}^{2} /\sigma_{{\text{M}}}^{2}$ values in non-healthy areas increased with the progression of fibrosis. This result indicates that $\sigma_{{\text{H}}}^{2} /\sigma_{{\text{M}}}^{2}$ corresponds to echo signals from fibrotic tissue.

**Fig. 7:**
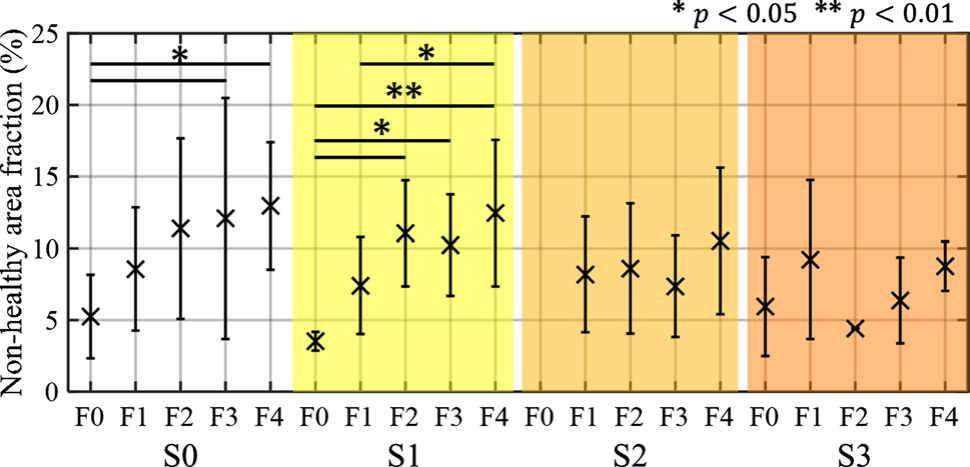
Mean values and standard deviation of non-healthy area fraction calculated using the HLSF method for each fatty liver grade and level of fibrosis progression. In the multiple comparison test, there was a statistically significant difference between F0 and F3 (*$p<0.05$) and between F0 and F4 (*$p<0.05$) in the S0 liver. In the S1 livers, there was a statistically significant difference among F0 and F1–F4 (*$p<0.05$ or **$p<0.01$) livers and between F1 and F4 (*$p<0.05$)

Figure 7 shows the mean value and standard deviation of the non-healthy area fraction for each level of fibrosis progression (F0–F4) and fatty liver grade (S0–S3). The vertical axis is the non-healthy area fraction. In S0 livers without lipid droplets (F0), the mean value of the non-healthy area fraction was 5.2%. In contrast, as fibrosis progressed (F1–F4) in S0 livers, the mean values were 8.6% (F1), 11.4% (F2), 12.1% (F3), and 13.0% (F4), which were all higher than for F0. In the fatty liver group, the mean value in S1 livers increased as fibrosis progressed. The mean value of F0 in S1 livers was 3.5%, whereas the mean values in F1 to F4 livers were more than twice that in F0. ANOVA confirmed a significant difference ($p<0.001$). In S0 livers, there was a significant difference in a multiple comparison test between non-healthy fraction of F0 livers and that of the F3 ($p=0.030$) and F4 ($p=0.021$) livers. In S1 livers, a significant difference in non-healthy area fraction was found between F0 and F2 ($p=0.019$), F3 ($p=0.023$) and F4 ($p=0.007$), and F1 and F4 ($p=0.024$). S2 and S3 did not increase with fibrosis, showing a different trend to S0 and S1.

## Discussion

HLSF and the MRA model were used to estimate the amount of fat and fibrous tissue for quantitative evaluation of NASH liver structure, which has characteristics of both fibrotic and fatty liver, and the evaluation accuracy was examined in livers with a mixture of fat and fibrous tissue. This study had potential limitations. All recruited patients shared the same etiology as they were positive for HBV and/or HCV. This should be recognized as a limitation of the study. The enrolled population did not cover all the possible combinations of fatty liver grade and METAVIR score. Although fat mass was assessed with liver biopsy in this study, magnetic resonance spectroscopy (MRS) is a more reproducible method of measuring liver fat mass than liver biopsy. This difference potentially affected the accuracy of fat mass assessment. The clinical data used in this study were quantized at 8 bits and also had the limitation of low spatial resolution. These characteristics of the measurement system may have affected the accuracy of the analysis.

The relationship was investigated between non-MRA fraction (which is considered to reflect the characteristics of aggregated lipid droplets) and fat mass. The characteristics of the moments judged as non-MRA were significantly different from those of fibrous tissue judged as MRA in Fig. 3c. In a previous study that used the DN model to evaluate fat characteristics, numerical simulations and examination of clinical data showed that the characteristics of aggregated lipid droplets in the frequency band used by clinical equipment (3–5 MHz) agreed with the moment characteristics of the amplitude envelope judged as non-MRA [20]. Several previous studies have also shown that non-MRA represents signals from lipid droplet aggregates. In Fig. 4a, the non-MRA fraction and fat mass were found to be weakly positively correlated. However, some livers with fat mass < 5% were found to have a non-MRA fraction of 10%, which is similar to that in fatty liver (fat mass > 5%). In such cases, tissue characteristics that do not correspond to aggregated lipid droplets may be observed as non-MRA. Although the luminal structure of normal liver can be approximated by a homogeneous distribution of scatterers, echoes in some tissues (such as blood vessels) are detected as if they were structures. Therefore, we consider that these tissues are detected as noise components. Based on these results, we compared the non-MRA fraction among fatty liver grades in patients without fibrosis (F0). The non-MRA fraction tended to increase with the progression of fatty liver disease, and significant differences were observed between S0 and S3, and between S1 and S3, in Fig. 4b. These findings indicate that the non-MRA fraction may include noise components such as blood vessels; however, differences in fat mass were significantly reflected in the non-MRA fraction. In addition, the non-MRA fraction identified on the basis of moments could be used as an indicator to assess fat mass. In other words, the present findings suggest the possibility of identifying and assessing moderate and severe (S2 and S3) fatty liver using non-MRA fraction.

In the evaluation of fibrosis progression, HLSF combined with MRA model parameters was proposed as a new analytical method. In Fig. 6a, the liver fibrosis parameter ${\alpha }_{\mathrm{H}}$ varied from 0 to 0.8 in the HLSF, which is not typical of normal liver. In contrast, $\sigma_{{\text{H}}}^{2} /\sigma_{{\text{M}}}^{2}$ tended to increase with fibrosis progression, indicating that the polar coordinate probability distribution of fibrosis parameters extends in the radial direction. The relationship between the probability distribution of fibrosis parameters and fibrosis progression was similar to that reported in previous studies that used the MRA model [21, 23]. Therefore, the combination of HLSF and the MRA model could visualize areas in which the characteristics differed from those of normal liver tissue in Fig. 6b, c. In addition, the non-healthy areas extracted outside the HLSF are considered to be strongly influenced by the characteristics of fibrotic tissue. To investigate the relationship between the non-healthy area fraction calculated using the proposed method and progression of fibrosis, Fig. 7 shows the trends in fibrosis progression and the non-healthy area fraction in S0 livers with low fat content. The non-healthy area fraction was higher in F1–F4 livers than in F0 livers with no fibrous tissue, and the percentage tended to increase as fibrosis progressed. The results indicated that the non-healthy areas extracted with HLSF corresponded to fibrotic tissue. However, there was no significant difference in the non-healthy area fraction between F0 and F1, or between F0 and F2. The reason for the lower detection accuracy may be that changes in tissue structure that occur in advanced fibrosis are not reflected outside the HLSF in early hepatitis.

Fat mass estimation of the non-MRA fraction and fibrosis progression estimation of non-healthy area fraction were applied to the case of mixed lipid droplets and fibrotic tissue, such as NASH liver. We evaluated the relationship between fat mass and non-MRA fraction in patients with advanced fibrosis. As shown Fig. 4b, in S0 livers, there was little change in the non-MRA fraction at each fibrosis progression, and no effect of fibrosis progression was observed, whereas in S1 to S3 livers, the relationship between fibrosis progression and the non-MRA fraction showed that the non-MRA fraction tended to decrease with fibrosis. This tendency was particularly pronounced in S2 and S3 livers, suggesting that the ability to detect the characteristics of aggregated lipid droplets may decrease with progression of fibrosis. However, when compared among patients with the same level of fibrosis progression, S2 and S3 showed higher values than S0 and S1. This result suggests that different fatty characteristics can be detected at the same level of fibrosis progression, although detectability decreases as fibrosis progresses.

We evaluated the relationship between fibrosis progression and non-healthy area fraction in patients with fatty liver. In Fig. 7, the non-healthy area fraction tended to increase with fibrosis in S1 livers. Comparing the results among the levels of fibrosis progression, significant differences in the non-healthy area fraction were found between F0 and F2–F4, and also between F1 and F4. However, in S2 and S3 livers, the non-healthy area fraction did not increase with fibrosis. These results suggest that patients with S1 livers can be evaluated for fibrosis without the influence of lipid droplets, whereas in those with S2 and S3 livers, it may not be possible to distinguish fibrous tissue characteristics well due to the influence of lipid droplets. The reason the proposed method does not work well in S2 and S3 livers appears to be that the increase in lipid droplets causes a deviation from the tissue structure assumed by the MRA model. In S2 and S3 livers, the lipid droplets are densely distributed with high echo scatterers, and thus have a homogeneous scatterer distribution structure even in cases of advanced fibrosis, compared to those without lipid droplets. This tissue structure deviates from that of fibrosis tissue considered in the MRA model. As mentioned as a limitation of the dataset, the low resolution of the measurement system results in spatial smoothing. Therefore, in cases with a high degree of fatty liver, even if fibrotic structures are included in the received signal, the characteristics of the fibrotic structures are considered to be squashed when the signals are converged as line signals in the receiving beam. Therefore, the signal from lipid droplets masks the signal component of fibrotic tissue in moderate and severe fatty liver, and the distribution characteristics of the echo signal become close to the Rayleigh distribution. Fibrotic tissue characteristics are distributed outside the HLSF; however, in patients with fatty liver, they appear to be distributed within the HLSF or are not reflected in fibrosis parameters due to the influence of lipid droplets.

In livers with mixed fat and fibrous tissue, grade S1 tended to have higher values than grade S0 in fat mass estimation based on non-MRA fraction. Estimation of fibrosis progression by combining the HLSF and MRA models showed that cases of early fatty liver (S1) showed significantly higher values with progression of fibrosis. These results suggest the possibility of simultaneous assessment of fat mass and fibrosis progression in early fatty liver. The conventional method of fatty liver assessment via the attenuation coefficient using clinical ultrasound can identify moderate and severe fatty liver. However, the attenuation coefficient varies and does not have high accuracy for detecting early fatty liver [35]. Compared to conventional techniques, the proposed method has the advantage of being able to detect fatty liver in early stages and evaluate fibrosis progression related to cirrhosis and hepatocarcinoma. The ability to assess fibrosis progression that determines the prognosis of patients with fatty liver is clinically useful. However, it was challenging to use the non-MRA score and non-healthy area fraction by themselves in the simultaneous estimation of fat mass and fibrosis progression, because no significant difference was confirmed with the proposed method in moderate and severe fatty liver. In the evaluation of patients with moderate fibrosis and fat content, it is necessary to examine the combined use of fat assessment via the attenuation coefficient and liver stiffness assessment index via elastography in addition to the proposed method. In addition, the fact that the clinical data we used on this study were quantized at 8 bits, which is lower than the quantization number used in recent abdominal ultrasound examinations (12–16 bits), may affect the accuracy of the analysis.

## Conclusion

In this study, we investigated the possibility of liver evaluation by discrimination between lipid droplets and fibrous tissue using amplitude envelope statistical analysis. Determining the number of components in the MRA model using moments enabled extraction of the characteristics of aggregated lipid droplets as non-MRA. The non-MRA fraction-based fat mass estimation suggested that fatty liver identification was possible. The evaluation of fibrosis progression by combining the MRA model and HLSF suggested that the non-healthy area fraction extracted via HLSF increased in response to fibrosis, and assessment of fibrosis using this method could identify moderate and severe fibrosis. In the fatty liver, this method suggested that quantitative evaluation of fibrosis progression was possible in the early fatty liver group. In moderate to severe fatty liver, however, the lipid droplet characteristics dominated those of fibrous tissue, and it was therefore difficult to evaluate the two tissues separately. These results suggest that it is possible to evaluate the degree of fibrosis progression in early-stage fatty liver. Therefore, the present results suggest the potential of our method for assessing progression of fibrosis in early fatty liver. The results suggested the possibility of simultaneous evaluation of fat and fibrous tissue, which had been challenging with conventional methods.
