# Correction: Modified multi-Rayleigh model-based statistical analysis of ultrasound envelope for quantification of liver steatosis and fibrosis

**DOI:** 10.1007/s10396-024-01454-8

**Published:** 2024-04-05

**Authors:** Yuki Ujihara, Kazuki Tamura, Shohei Mori, Dar-In Tai, Po-Hsiang Tsui, Shinnosuke Hirata, Kenji Yoshida, Hitoshi Maruyama, Tadashi Yamaguchi

**Affiliations:** 1 Graduate School of Science and Engineering, Chiba University, 1-33 Yayoicho, Inage, Chiba, 2638522 Japan; 2 Preeminent Medical Photonics Education and Research Center, Hamamatsu University School of Medicine, Hamamatsu, Shizuoka 4313192 Japan; 3 Graduate School of Engineering, Tohoku University, Sendai, Miyagi 9808579 Japan; 4 Department of Gastroenterology and Hepatology, Chang Gung Memorial Hospital at Linkou, Taoyuan, 33305 Taiwan; 5 Division of Pediatric Gastroenterology, Department of Pediatrics, Chang Gung Memorial Hospital at Linkou, Taoyuan, 33305 Taiwan; 6 Department of Medical Imaging and Radiological Sciences, College of Medicine, Chang Gung University, Taoyuan, 33305 Taiwan; 7 Center for Frontier Medical Engineering, Chiba University, 1-33 Yayoicho, Inage, Chiba, 2638522 Japan; 8 Department of Gastroenterology, Juntendo University, Bunkyo, Tokyo, 1138421 Japan

**Correction: Journal of Medical Ultrasonics (2024) 51:5–16** 10.1007/s10396-023-01354-3

The article “Modified multi-Rayleigh model-based statistical analysis of ultrasound envelope for quantification of liver steatosis and fibrosis”, written by Yuki Ujihara, Kazuki Tamura, Shohei Mori, Dar-In Tai, Po-Hsiang Tsui, Shinnosuke Hirata, Kenji Yoshida, Hitoshi Maruyama and Tadashi Yamaguchi, was originally published Online First without Open Access. After publication in volume 51, issue 1, pages 5–16 the author decided to opt for Open Choice and to make the article an Open Access publication. Therefore, the copyright of the article has been changed to © The Author(s) 2023 and the article is forthwith distributed under the terms of the Creative Commons Attribution 4.0 International License, which permits use, sharing, adaptation, distribution and reproduction in any medium or format, as long as you give appropriate credit to the original author(s) and the source, provide a link to the Creative Commons licence, and indicate if changes were made. The images or other third party material in this article are included in the article’s Creative Commons licence, unless indicated otherwise in a credit line to the material. If material is not included in the article’s Creative Commons licence and your intended use is not permitted by statutory regulation or exceeds the permitted use, you will need to obtain permission directly from the copyright holder. To view a copy of this licence, visit http://creativecommons.org/licenses/by/4.0.

The original article has been corrected.

